# Rapid abdominopelvic MR imaging in the emergency department: establishing a program and addressing the challenges

**DOI:** 10.1007/s00247-024-06004-5

**Published:** 2024-07-23

**Authors:** Rama S. Ayyala, Jonathan R. Dillman, Jean A. Tkach, Andrew T. Trout

**Affiliations:** 1 Department of Radiology, Cincinnati Children’s Hospital Medical Center, 3333 Burnett Avenue, Cincinnati, OH 45229 USA; 2 Department of Radiology, University of Cincinnati College of Medicine, Cincinnati, OH USA; 3 Department of Pediatrics, Cincinnati Children’s Hospital Medical Center, Cincinnati, OH USA

**Keywords:** MRI, Pediatric, Emergent

## Abstract

Utilization of magnetic resonance imaging (MRI) in the pediatric emergency room or urgent care setting for abdominopelvic indications has been increasing. The creation and implementation of rapid urgent MRI programs can have various challenges. The purpose of this article is to describe a framework for the creation of a rapid urgent abdominopelvic MRI program in the pediatric emergency room setting.

## Introduction

Utilization of magnetic resonance imaging (MRI) in the pediatric emergency room or urgent care setting for abdominopelvic indications has been increasing. For example, MRI for evaluation of suspected acute appendicitis is performed at many pediatric radiology departments nationally. The creation and implementation of rapid urgent MRI programs are fraught with challenges. The purpose of this article is to describe a framework for the creation of a rapid urgent abdominopelvic MRI program in the pediatric emergency room setting. A single institution’s experience will be utilized to discuss the steps needed to create a program and highlight barriers and challenges with tips on how to manage them.

## Why MRI in the emergency department?

Ultrasound (US) has long been the first-line imaging modality of choice in the emergent room or urgent care setting for many causes of abdominopelvic pain in children. It is relatively accessible, does not require sedation, can be performed portably, does not involve ionizing radiation, and is less expensive (to both patient and provider) than other imaging modalities such as computed tomography (CT) and MRI. However, US can have diagnostic limitations in certain situations, such as in patients with larger body habitus or copious bowel gas. Ultrasound is also operator dependent and is optimally performed by trained dedicated pediatric sonographers to ensure adequate, accurate imaging. Although CT has long been an alternative and second-line imaging modality for pediatric abdominopelvic pain in the emergent setting, MRI is emerging as a preferred alternative in an increasing array of situations, when available. As a result, studies have shown that the use of MRI in the emergent setting has increased over time for all indications, including abdominopelvic imaging [1, 2].

The most common indication for abdominopelvic MRI in the pediatric emergency room or urgent care setting is for suspected acute appendicitis. There is a large body of literature showing the diagnostic accuracy of MRI for appendicitis in both adults and children [3, 4]. A recent survey among members of the Society of Chiefs of Radiology at Children’s Hospitals (SCORCH) with 39 respondents showed that 21 institutions currently perform MRI appendicitis [5]. Other institutions endorsed the desire to start a program; however, they cited challenges that first needed to be overcome, including MRI availability, adequate staffing of the MRI scanner, getting buy-in from various stakeholders, potential cost differential, and need for a broad change in culture in urgent abdominopelvic imaging.

Other indications for abdominopelvic MRI in the emergent setting have been described in the literature. Rapid MRI examination can be performed for atraumatic pediatric abdominal pain in the emergent setting for detecting a variety of diagnoses other than appendicitis [6, 7]. MRI has also been used in the emergent setting to assess for postoperative abscess [8, 9]. At the author’s institution, MRI is used as first-line imaging in certain clinical scenarios to evaluate for ovarian torsion [10]. Additional literature has shown that MRI can be utilized to assess for other gynecological pathologies (e.g., ovarian mass, congenital Mullerian anomalies) in children in the emergent setting that may be difficult to delineate on ultrasound alone [11]. In adults, there are studies showing the utilization of MRI for hepatobiliary and renal indications [12, 13]; however, no similar studies in pediatric patients have been performed to date.

## Steps for creating a program for urgent body MRI

Adoption of MRI for pediatric abdominopelvic imaging in the emergency room or urgent care setting can be challenging; however, using a stepwise approach can address potential barriers. At the author’s institution, a rapid MRI protocol is currently performed to evaluate for suspected appendicitis and ovarian torsion in the emergent setting [10, 14]. Through the experience of creating these programs, five key steps for establishing an effective rapid urgent MRI program were identified: (1) discussion and buy-in from the involved stakeholders, (2) creating appropriate MRI protocol(s), (3) determining appropriate billing codes, (4) disseminating appropriate education about the program and examination to the various involved groups, and (5) implementation of the program with regular quality assessment (Table 1). Each step will be detailed with examples from the authors’ experience of creating rapid urgent MRI programs.

**Table 1:** Stepwise approach for establishing rapid MRI program for abdominopelvic indications in children

Five steps for establishing an effective rapid MRI program
1. Discussion and buy-in from involved stakeholders
2. Create appropriate MRI protocol
3. Determine appropriate MRI billing code
4. Creation and dissemination of education
5. Implementation and quality control of the program

### Discussion and buy-in from involved stakeholders

To obtain successful buy-in for a rapid urgent MRI program, it is critical to begin discussions with all potential stakeholders early in the process. For instance, creating an MRI appendicitis program requires early involvement and engagement of the emergency department and pediatric surgery teams, as well as with members of one’s own radiology department. These discussions should involve presentation of the data supporting use of MRI for appendicitis and the rationale for moving away from CT to MRI, and should address concerns and questions. Similarly, for our MRI ovarian torsion program, discussions were held with the emergency department and pediatric gynecology teams to present the benefits of MRI over ultrasound for torsion (e.g., no need to fill the urinary bladder), and to discuss how ovarian torsion is diagnosed on MRI, with specific focus on not needing Doppler imaging findings on pelvic US for accurate diagnosis [15, 16]. Aligning the involved clinical teams with the purpose of the MRI examination, and how it compares to other imaging modalities, is imperative to have successful buy-in to the program and to guide appropriate utilization. Once this is established, it is helpful to create imaging pathways to assist ordering providers determine when to order an MRI. This is critical to ensure that imaging is utilized appropriately and to avoid unnecessary imaging, which can be costly and a waste of resources.

Radiology operations meetings to discuss the availability of the MRI scanner and appropriate staffing are necessary to ensure optimal workflow. At the authors’ institution, an MRI scanner is located in the emergency department with dedicated staffing during available hours. This allows for ease of transport to and from the emergency department and for urgent examinations to be performed promptly. In many departments, the MRI scanners may be located remotely from the emergency department; therefore, safe methods for patient transport need to be created as part of the workflow. At some institutions, the MRI scanners may be shared with adult radiology, which may limit the time available for urgent pediatric cases. MRI may not be available 24 h, as at the author’s institution, or at all times due to shared use; therefore, alternative imaging modalities such as CT must be in place for when MRI is not available. Alternative modalities already exist for urgent imaging indications and are established as the standard of care. Therefore, the lack of 24-h or guaranteed availability of MRI should not be a limitation in implementing a program for rapid urgent MRI.

### Creating the appropriate MRI protocol

Full-length abdominopelvic MRI protocols such as those performed for outpatient indications are often long (> 30 min), and many involve administration of intravenous gadolinium-based contrast material. MRI protocols for abdominopelvic imaging in the emergent setting are better built as screening examinations to evaluate for specific diagnostic questions with a relatively short scan duration. Therefore, abbreviated MRI examinations with a limited number of targeted sequences, ideally performed free breathing and without the administration of intravenous contrast, should be employed.

Currently, there is significant variability in what constitutes an abbreviated (or rapid) MRI examination. A survey of pediatric radiology departments in North America demonstrated that MRI appendicitis protocols varied widely across institutions. The number of sequences performed ranges from 3 to 8, with some utilizing contrast material, while others performed a noncontrast examination [5]. A recent Society for Pediatric Radiology (SPR) consensus paper on abbreviated MRI for appendicitis stated that the most important sequences for evaluation of appendicitis are T2-weighted images in multiple planes, with and without fat saturation, and intravenous contrast is not necessary. Diffusion-weighted imaging was suggested as an optional sequence but was indicated not to be essential for adequate evaluation [17].

**Fig. 1:**
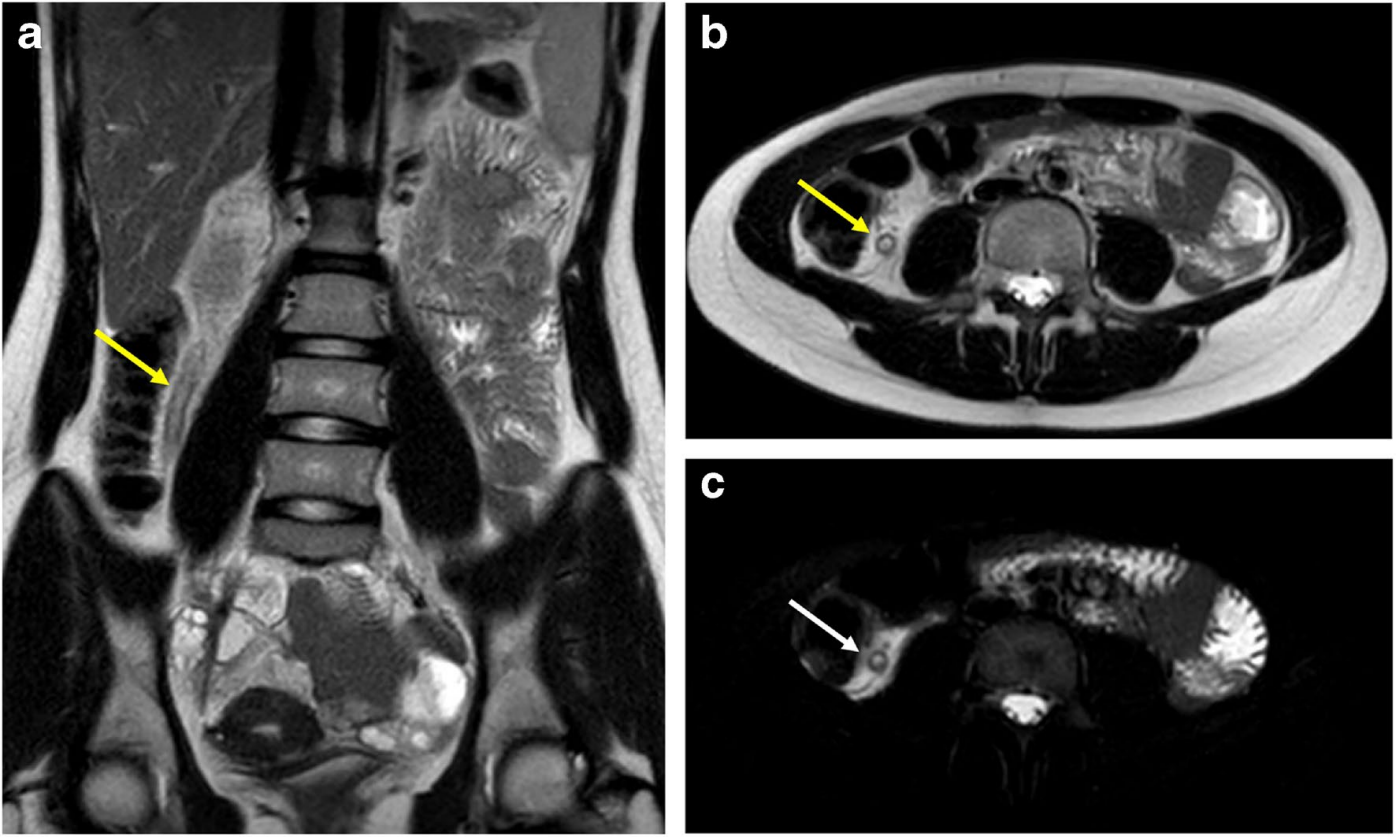
Acute appendicitis. 15-year-old girl with right lower quadrant pain for 1 day. **A** Coronal T2 SSFSE image and (**B**) axial T2 SSFSE without fat saturation image demonstrate dilated appendix in the right lower quadrant, medial to the cecum (yellow arrows). **C** Axial T2 SSFSE with fat saturation shows periappendiceal fat inflammatory changes (white arrow). Surgery and pathology confirmed acute non-complicated appendicitis

**Fig. 2:**
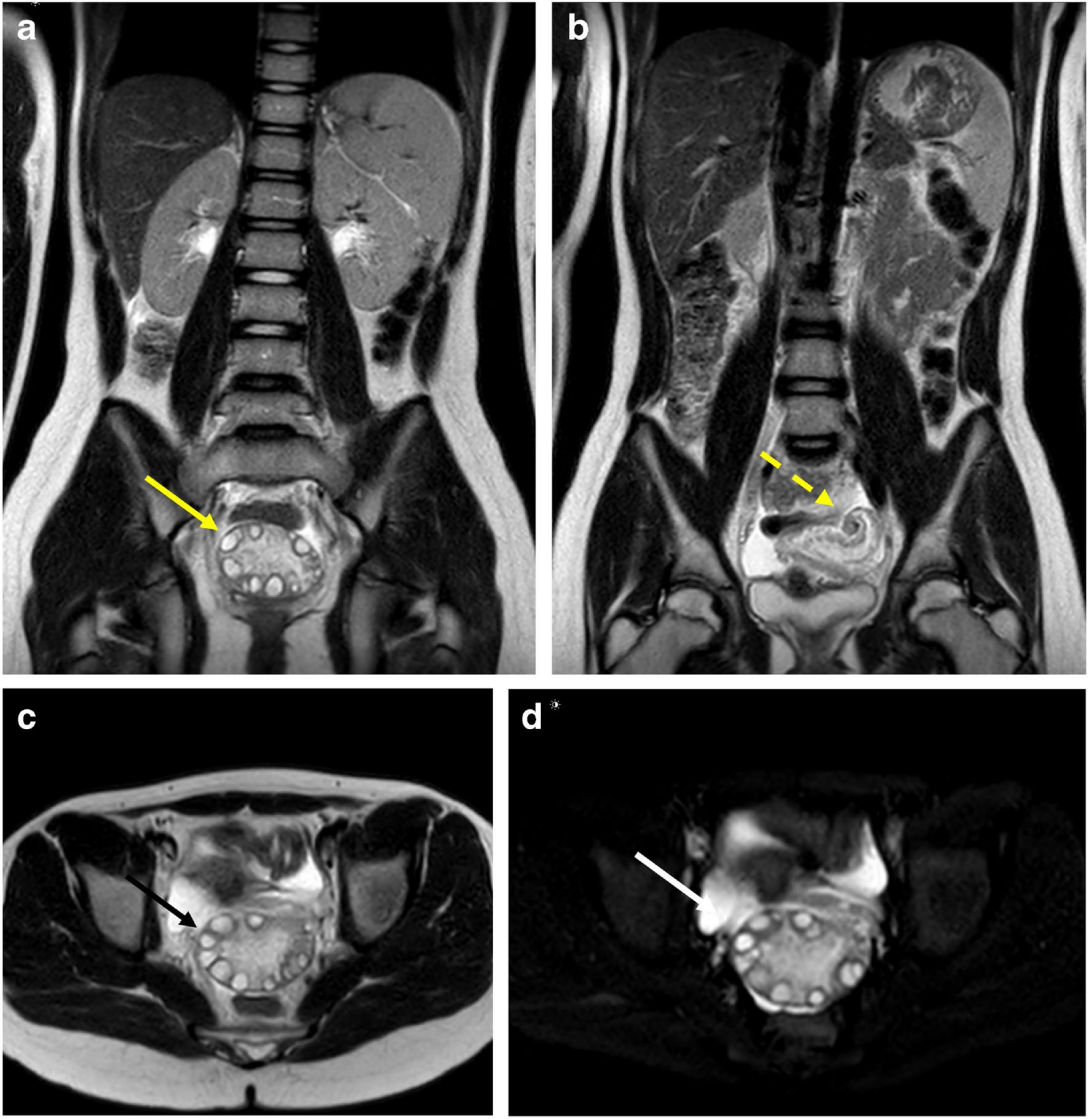
Ovarian torsion. 11-year-old girl with 2 weeks of intermittent left side abdominal pain, acutely worse for 1 day with emesis. **A** and (**B**) Coronal T2 SSFSE images demonstrate enlarged left ovary displaced to the midline, with peripheralization of follicles and edema of the stroma (yellow arrow), with a twisted left infundibulopelvic ligament (yellow dotted arrow). Axial T2 SSFSE (**C**) without fat saturation and (**D**) with fat saturation show the enlarged left ovary displaced posterior to the uterus (black arrow), with surrounding fluid (white arrow). Surgery confirmed left ovarian torsion with 720° twisting of the left infundibulopelvic and utero-ovarian ligaments

At the author’s institution, a rapid MRI protocol including only T2-weighted sequences has been implemented for evaluation of MRI appendicitis and ovarian torsion (same protocol for both indications) (Table 2). The examination does not require intravenous contrast material and is entirely free breathing and without respiratory navigation/triggering. The median scan time (first image acquired to last image acquired) is 5 min, facilitating patient tolerance, as well as workflow and prompt diagnosis. This protocol has been shown to be feasible and effective for accurate diagnosis of both appendicitis and ovarian torsion [10, 14] (Figs. 1 and 2).

**Table 2:** Representative rapid MRI protocol for acute appendicitis and ovarian torsion at the authors’ institution. All sequences are acquired absent any form of respiratory compensation. Spatial coverage is set to include the anatomy from just above the gall bladder through the pubic symphysis. *SSFSE* single-shot fast spin echo, *FS* fat saturation, *SENSE* sensitivity encoding, *SPAIR* spectral attenuated inversion recovery

Rapid MRI protocol Acquisition parameters	Coronal T2W SSFSE	Axial T2W SSFSE	Axial T2W SSFSE w FS
Field-of-view (mm)	400	260	260
Acquired in-plane spatial resolution (mm × mm)	1.4 × 1.4	1.4 × 1.4	1.5 × 1.5
Slice thickness/gap (mm/mm)	5/0	5/0	6/1
Number of slices	~ 34	~ 60	~ 51
Repetition time	“Infinite”; single shot	“Infinite”; single shot	“Infinite”; single shot
Echo time	80	80	80
Flip angle (degrees) (excitation/refocusing)	90/135	90/145	90/135
Half scan factor (aka half Fourier)	0.7	0.75	0.63
Turbo spin echo factor	79	59	105
Acceleration method	SENSE	Compressed SENSE	SENSE
Acceleration factor	2.75	2.5	1.3
Number of signal averages	1	1	1
Fat saturation	None	None	SPAIR
Scan time (s)	16.4	24.3	33.0

As with every step in the process of developing a successful MRI program, involvement of critical team members with continued communication is important. In the development of a MRI protocol, it is important to engage the MRI technologists to get input on how to tailor an examination optimally for the emergent setting, especially for children who will not have sedation. Medical physicists can help optimize the MRI sequences for both image quality and faster scan times. Lastly, discussing the protocol with radiologists and illustrating example examinations for review can help determine the best overall protocol for fast yet accurate diagnosis.

### Determining the appropriate MRI billing code

In the United States, there is no dedicated billing code for a rapid abdominopelvic MRI, or for MRI performed for specific diagnoses such as appendicitis. Under current billing paradigms, the type of sequences performed, the anatomic coverage included in the examination, and if intravenous contrast material is administered are used to determine the appropriate billing codes. There is no accounting for the number of sequences performed or the duration of the examination. The recent survey performed of pediatric radiology departments in North America showed variability in the different billing codes utilized for MRI appendicitis examinations. The most common billing codes are MRI abdomen/pelvis without intravenous contrast material and MRI pelvis without intravenous contrast material [5]. Given that rapid MRI examinations include fewer sequences than typical MRI examinations billed with these codes, a limited modifier can be attached by the billing entity to potentially decrease the associated reimbursement. The actual cost associated with performing a rapid MRI is significantly less than the cost for examinations with billing codes typically used for these examinations [18]. The creation of a specific billing code for limited MRI needs to be considered to decrease the cost associated with rapid MRI examinations, which will aid in more widespread adoption and establishment of more novel indications.

### Creation and dissemination of education

**Fig. 3:**
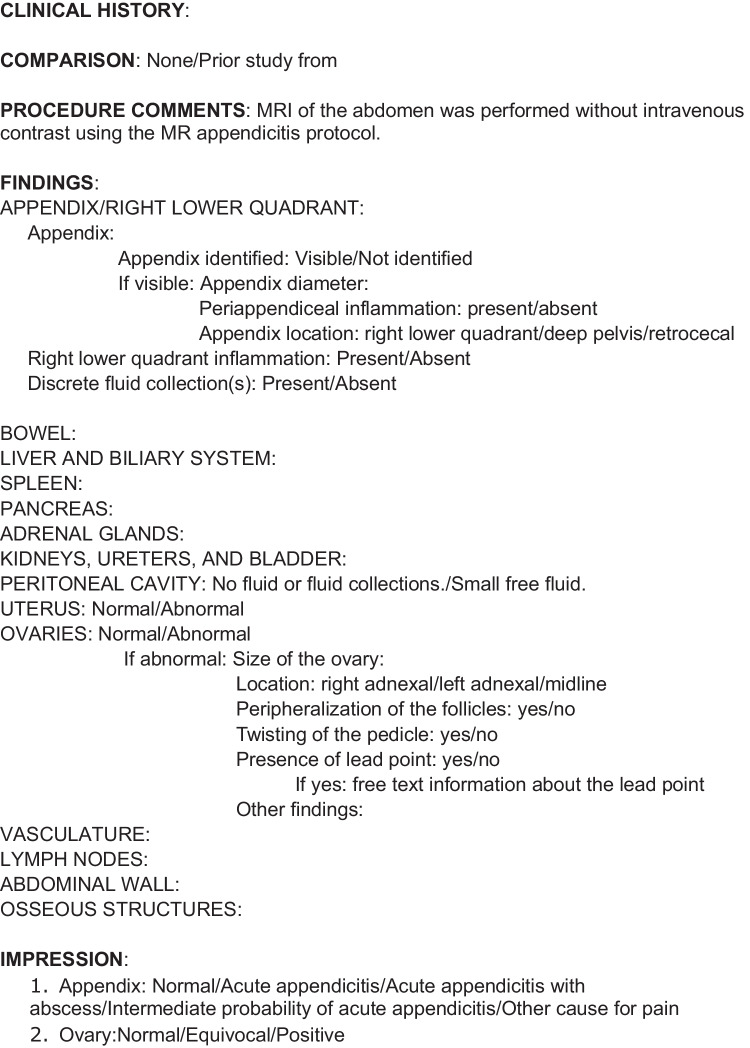
Structured reporting. Example structured report used at the authors’ institution for MRI appendicitis and MRI ovarian torsion examinations

After finalizing the examination protocol, operational workflow, and billing mechanisms, education regarding the overall rapid MRI program is important for all the involved groups. This includes radiologists, radiology trainees, technologists, and clinical stakeholders. For the radiologists and radiology trainees, educational sessions reviewing the background for instituting MRI for a clinical indication and the specified MRI protocol, with justification for the various sequences, can provide an explanation for the why behind the change in imaging algorithms. Subsequently, outlining the workflow guidelines (availability times, exclusionary criteria for patients in MRI, etc.) is important for successful operationalization of the program. The creation and implementation of a structured radiology report for the specific clinical indications are critical to ensure standardized reporting and communication of necessary information for clinical management. Implementation of standardized reports with fixed impressions can also reduce unnecessary hedging during the early phases of program implementation or when onboarding new readers (Fig. 3). Reviewing example cases to illustrate imaging findings can facilitate familiarity with the examination and typical imaging findings. It is important to review not only the imaging findings of a specific diagnosis to illustrate false positive and false negative examinations, but also additional examinations with imaging findings of potential alternative diagnoses. Similar educational sessions should be conducted with MRI technologists not only to emphasize the protocol, but also to review tips for optimal scanning and troubleshooting tricks and strategies/paradigms to help in difficult situations (i.e., patients with larger body habitus, uncooperative patients, etc.).

Educational sessions with the clinical stakeholders need to be tailored with information for the specific indication. For MRI appendicitis, discussions at regular staff meetings for the emergency department and pediatric surgery faculty helped our site introduce and review the program as well as serve as a forum to discuss concerns or questions. Similar discussions have been held with the gynecology team regarding the MRI ovarian torsion program. Educational sessions using patient cases have been regularly conducted with the trainees in various clinical departments to highlight important educational points, discuss difficult clinical scenarios, and provide feedback to both the clinical and radiology teams for future cases.

### Implementation and quality control of the program

After establishing a rapid MRI program, continued monitoring of the program and examinations performed serves to assess quality of the examinations, diagnostic performance, and appropriate utilization. At the authors’ institution, a lead MRI technologist and physicist participate with a lead physician in the quality review of rapid MRI examinations for appendicitis and ovarian torsion. This ongoing review allows iterative changes to improve the process and address any issues that arise. Typical review at the author’s institution includes assessment of image quality, interpretation of the examination, associated surgical and pathology findings (if applicable), and any follow-up imaging. Cases with missed findings or important learnings are shared with the radiology faculty and trainees. Image quality issues are reviewed with the MRI technologists and physicists to improve future examinations. Inappropriate utilization of the imaging pathways is shared with the clinical teams to provide continued feedback and education of the ordering providers as well as to maintain engagement in the program. Although this process is time intensive, it is vital to ensure an implemented program continues to work effectively.

### Future directions of rapid urgent MRI for body imaging

**Fig. 4:**
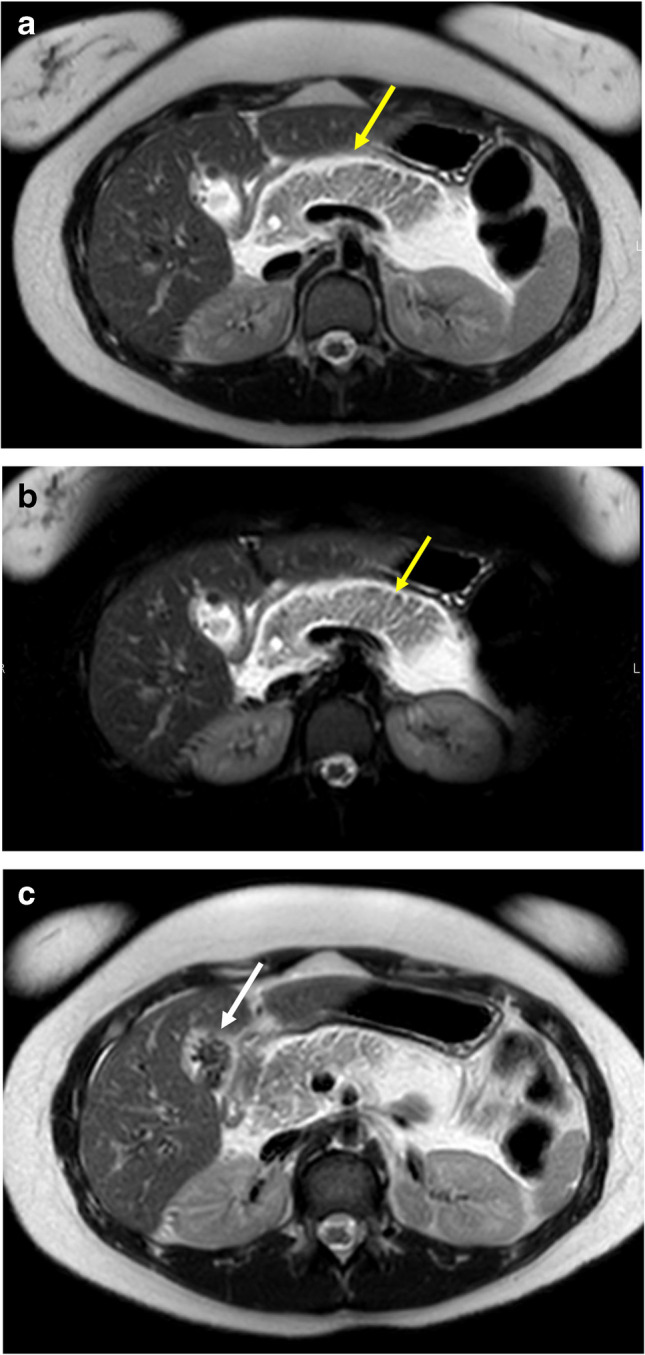
Alternative diagnosis: pancreatitis. 14-year-old girl with severe abdominal pain for 1 day. **A** Axial T2 SSFSE without fat saturation and (**B**) with fat saturation images demonstrate enlarged edematous pancreas, with peripancreatic inflammatory changes and fluid (yellow arrows). **C** Additional axial T2 SSFSE without fat saturation image demonstrates gallstones in the gallbladder (white arrow), which was the etiology of the acute interstitial pancreatitis

MRI is well established for diagnosis of appendicitis in pediatric patients, and additional studies have shown that MRI can be used for detecting ovarian torsion as well as other gynecological etiologies of pelvic pain [10, 14]. An abbreviated MRI might be used to detect other diagnoses as well, such as pancreatitis and enteritis [3, 14] (Fig. 4).

Rapid MRI has the potential to be used as a screening tool to evaluate for causes of nonspecific abdominal and pelvic pain in the emergent setting for children. It is possible that earlier use of MRI in the emergency department encounter might improve patient throughput and even lower healthcare costs in the long run. Further work is needed to confirm these suppositions and address the challenges and barriers discussed in this review in order to make MRI more widely available.

## Conclusion

With the increasing utilization of MRI in the emergency room or urgent care setting for pediatric patients, guidance on how to create successful and sustainable programs is needed. A systematic, stepwise approach can be used to implement use of MRI for various abdominopelvic indications in the emergency room or urgent care setting. Clear algorithms and structure, as well as appropriate education and communication with the involved stakeholders, are the foundation to provide optimal patient care while maintaining efficient operational workflow.

## Data Availability

All authors ensure the data and materials presented comply with field standards.

## Code availability

N/A.
